# Immunogenic and protective properties of GP5 and M structural proteins of porcine reproductive and respiratory syndrome virus expressed from replicating but nondisseminating adenovectors

**DOI:** 10.1186/1297-9716-44-17

**Published:** 2013-03-11

**Authors:** Elodie Roques, Aurélie Girard, Marie-Claude St-Louis, Bernard Massie, Carl A Gagnon, Martin Lessard, Denis Archambault

**Affiliations:** 1 Department of Biological Sciences, University of Québec at Montréal, Succursale Centre-Ville, P.O. Box 8888, Montréal, Québec, H3C 3P8, Canada; 2 Biotechnology Research Institute, National Research Council of Canada, 6100 Royalmount Avenue, Montréal, Québec, H4P 2R2, Canada; 3 Adjunct professor, Department of Microbiology and Immunology, University of Montréal, Succursale Centre-Ville, P.O. Box 6128, Montréal, Québec, H3C 3J7, Canada; 4 Groupe de recherche sur les maladies infectieuses du porc, Faculty of Veterinary Medecine, University of Montréal, 3200 rue Sicotte, St-Hyacinthe, Québec, J2S 7C6, Canada; 5 Dairy and Swine Research and Development Centre, Agriculture and Agri-Food Canada, 2000 College Street, Sherbrooke, Québec, J1M 1Z3, Canada

## Abstract

Porcine reproductive and respiratory syndrome virus (PRRSV) is responsible for significant economic losses in the porcine industry. Currently available commercial vaccines do not allow optimal and safe protection. In this study, replicating but nondisseminating adenovectors (rAdV) were used for the first time in pigs for vaccinal purposes. They were expressing the PRRSV matrix M protein in fusion with either the envelope GP5 wild-type protein (M-GP5) which carries the major neutralizing antibody (NAb)-inducing epitope or a mutant form of GP5 (M-GP5m) developed to theoretically increase the NAb immune response. Three groups of fourteen piglets were immunized both intramuscularly and intranasally at 3-week intervals with rAdV expressing the green fluorescent protein (GFP, used as a negative control), M-GP5 or M-GP5m. Two additional groups of pigs were primed with M-GP5m-expressing rAdV followed by a boost with bacterially-expressed recombinant wild-type GP5 or were immunized twice with a PRRSV inactivated commercial vaccine. The results show that the rAdV expressing the fusion proteins of interest induced systemic and mucosal PRRSV GP5-specific antibody response as determined in an ELISA. Moreover the prime with M-GP5m-expressing rAdV and boost with recombinant GP5 showed the highest antibody response against GP5. Following PRRSV experimental challenge, pigs immunized twice with rAdV expressing either M-GP5 or M-GP5m developed partial protection as shown by a decrease in viremia overtime. The lowest viremia levels and/or percentages of macroscopic lung lesions were obtained in pigs immunized twice with either the rAdV expressing M-GP5m or the PRRSV inactivated commercial vaccine.

## Introduction

Porcine reproductive and respiratory syndrome (PRRS) emerged in the late 1980s in North America [1] and then later in Europe [2]. Since then, the disease has spread worldwide and become one of the most serious infections in the swine industry with an estimated loss of $ 664 million per year in the USA in 2011 [3]. PRRS is characterized by severe respiratory clinical signs associated with pneumonia in pigs of all ages and reproductive disorders in sows associated with late term abortion or premature farrowing and an increased number of stillborn piglets [4].

The causative agent of PRRS, the porcine reproductive and respiratory syndrome virus (PRRSV), belongs to the *Arteviridae* family which together with the *Coronaviridae* and *Roniviridae* families constitute the *Nidovirales* order [5]. The PRRSV genome is a positive, single-stranded, 5’-capped and 3’-polyadenylated mRNA molecule with a length of approximately 15 000 nucleotides (nt). It contains, in the direction 5’-3’, two large open reading frames (ORF), ORF 1a and 1b, which encode the viral replicase and represent approximately three-quarters of the genome, and seven smaller ORF designated 2a, 2b and 3 to 7 which express structural proteins termed GP2a, GP2b, GP3, GP4, GP5, M and N, respectively [6]. An additional structural protein, GP5a, exists and is encoded by an alternative ORF from the subgenomic viral mRNA encoding GP5 [7].

GP5 is a glycosylated envelope protein of approximately 25 kDa, carrying the major neutralizing epitope. An immunodominant region localized in the ectodomain of GP5 contains a so-called decoy epitope (amino acids (aa): 27–30 A/(V)LVN) [8]. Soon after infection, this epitope induces a strong non-neutralizing antibody (Ab) response and a delay in the production of neutralizing Ab (NAb) which generally appears after three weeks post infection [8,9]. However, the decoy epitope is not the only way for PRRSV to escape the host Ab response. GP5 contains several N-glycosylation sites located at or near the neutralizing epitope (aa 37 to 45: SHLQLIYNL) [10]. Abrogation of the N34 and N51 glycosylation sites within an infectious clone of PRRSV induces in the pig a faster and more efficient NAb response than the wild-type clone [11]. In contrast, it was reported that wild-type PRRSV strain induces a more rapidly and more strongly NAb response in infected pigs than natural mutant isolates carrying a disrupted N44 glycosylation site [12]. The GP5 protein is associated within the virion to the membrane protein M via disulfide bonds [13]. M is a non-glycosylated protein of approximately 19 kDa associated with a strong cellular immune response [14]. The use of GP5 either co-expressed or in fusion with M using various genetic vectors generates a better NAb response against GP5 than the use of GP5 alone [15-17].

Although there are several vaccines commercially available, these may have several pitfalls. Attenuated live vaccines present a risk of reversion to virulence [18] whereas inactivated vaccines may not confer optimal protection [19,20]. Because of this, several vaccine strategies have been developed against PRRSV. Most of these strategies rely on the use of DNA-based vaccines [21-26], transgenic plants [27,28], bacterial vectors [29-31], or viral vectors. Among the viral vectors used are non-replicative human [17,32-39] and canine adenoviruses [40], transmissible gastroenteritis virus [41], the modified strain of vaccinia virus [16], pseudorabies virus [15,42] and fowlpox virus [43]. Approximately half of these immunization studies were conducted in pigs that were then exposed to an experimental challenge, but only a few compared their efficacy with commercially-available vaccines [15,23,42]. In all these strategies a partial protection of immunized pigs was demonstrated [41].

Replicating but nondisseminating human adenoviruses serotype 5 (rAdV) have been developed. These adenovectors are devoid of functional protease (PS) gene, preventing the maturation process of capsid proteins and assembly of viral particles [44]. For this reason these vectors are considered safe because of their inability to spread among the population [45]. In addition, these vectors are functional in the E1A gene, allowing replication of the viral genome, and, thereof, expression of the proteins of interest to high levels within the inoculated host [45]. Finally, these adenovectors are of the human type rendering them very attractive for use in the pig which is fully permissive to the vector and does not have vector-specific pre-existing immunity [46].

In this study, the rAdV described above were used for the first time in pigs. rAdV were generated to express the PRRSV M protein in fusion with GP5 wild-type (M-GP5) or with a mutant form of GP5 (M-GP5m) that theoretically increases NAb response. Pigs were immunized with rAdV expressing GFP (negative control), M-GP5 or M-GP5m. Two additional groups were immunized once with rAdV expressing M-GP5m and boosted with recombinant bacterial GP5 (M-GP5m/rGP5 group) or twice with a PRRSV commercial inactivated vaccine (inactivated vaccine group) respectively. The results show that all immunized animals generated a GP5-specific Ab response. Pigs of the M-GP5m/rGP5 group developed the highest Ab response. Pigs that received the rAdV expressing M-GP5m or the inactivated vaccine twice showed the lowest viremia levels and/or percentages of macrocospic lung lesions after an experimental challenge with PRRSV.

## Material and methods

### Viruses, cells and synthetic genes

PRRSV IAF-Klop [33] and FMV09-1155278 (C.A. Gagnon, unpublished) strains were propagated in Dulbecco’s modified Eagle’s medium (DMEM; Invitrogen, Carlsbad, CA, USA) supplemented with 8% fetal bovine serum (FBS; PAA Laboratories, Inc., Etobicoke, Ontario, USA) at 37°C in a humidified atmosphere of 5% CO_2_[35] and titrated in MARC-145 cells using the Kärber method [47].

rAdV were propagated and titrated in the 293-PS-CymR cell line which is a cell clone derived from the HEK 293 cell line expressing the protease (PS) gene [44]. Cells were grown in DMEM supplemented with 8% FBS and 50 μg/mL cumate (Sigma-Aldrich, St. Louis, MO, USA) at 37°C in a 5% CO_2_ humidified atmosphere as described [48].

A549 cells were propagated in DMEM supplemented with 8% of FBS and maintained at 37°C in a humidified atmosphere of 5% CO_2_. Unlike the HEK 293 cells, these do not express proteins encoded by the adenovirus E1 gene and were used to confirm protein expression from the rAdV [44].

The codons most frequently used in *Sus scrofa* cells and demonstrated for their ability to increase the immune response in swine [35] were used to generate the GP5 (ORF5)- and M (ORF6)-encoding genes (synthesized through GeneArt services; Invitrogen) on the basis of the PRRSV IAF-Klop genome sequence [Genbank accession number: U64928].

### Cloning of GP5 wild-type or mutated sequences in fusion with the M sequence

Three mutations to increase the Ab and T cell immune responses specific to GP5 [22,25] were introduced by PCR in the GP5-encoding gene. The first mutation consisted in the replacement of the GCCCTGGTGAAC nucleotide (nt) sequence (aa 27–30: ALVN) by the TCTGGGTCTGGC nt sequence (aa 27–30: SGSG) to abolish the decoy ALVN epitope [8]. The second mutation introduced the 39 nt PADRE sequence (AKFVAAWTLKAAA) between residues 32 and 33 such that this sequence was localized between the abrogated decoy sequence and neutralizing epitope [25]. The third mutation replaced the AAC triplet (N) by the GCC triplet (A) to abolish the N51 glycosylation site [22]. The resultant protein produced from the mutated encoding gene was designated hereafter GP5m. GP5 wild-type- or GP5m-encoding sequences were fused by PCR to the 3’ terminal of the M gene with the insertion of the GTTACCACC (GTT) linker sequence between the M and GP5-encoding nt sequences [17]. The sequences were validated by DNA sequencing through the McGill University Sequencing Services (Montréal, QC, Canada).

### Construction of recombinant adenoviruses (rAdV)

The M-GP5- and M-GP5m-encoding sequences were inserted into the *Bgl*II site of the adenovirus transfer vector pAdenoVator-CMV5(CuO)-IRES-E1A [45]. The recombinant plasmids were rescued into the genome of the pAdeasyΔPS by homologous recombination in *E*. *coli BJ5183* cells (MP Biomedicals, Irvine, CA, USA) through electroporation (2.5 kV, 200 Ohms and 25 μF). The recombinant rAdV genome was confirmed by PCR and restriction enzyme analysis. To produce the rAdV, plasmids were linearized by *Pac*I digestion and 293-PS-CymR cells at 60% confluency were transfected with 2 μg of each plasmid (one well per plasmid of a 6-well tissue culture plate) using PolyFect transfection reagent (Qiagen, Valencia, CA, USA). The transfected cells were overlaid 24 h later with agarose (Invitrogen) (0.45% in DMEM supplemented with 5% FBS) and monitored daily until the appearance of viral plaques. After the confirmation of transgene expression from amplified viral clones, rAdV were produced at a large scale and purified by double cesium chloride gradient [45]. The infectious dose of rAdV was determined in 293-PS-CymR infected cells using the Kärber method and titers were expressed in tissue culture infectious dose 50 per mL (TCID_50_/mL).

### Western blot assay

A549 cells were seeded in 6-well tissue culture plates and infected with rAdV with a multiplicity of infection (MOI) of one. At 24 h post-infection, cells were lysed with lysis buffer (50 mM Tris, pH 7.4, 150 mM NaCl, 1% triton X-100 and EDTA-free protease-inhibitor cocktail (Roche, Indianapolis, IN, USA)) and total cell protein concentration was quantified with the DC protein assay kit (Bio-Rad, Mississauga, ON, CA, USA). For each sample, 20 μg of total cell extract was electrophoretically separated onto 12% SDS-PAGE and transferred to nitrocellulose membranes (Bio-Rad). The membranes were blocked in phosphate buffered saline (PBS) solution, pH 7.3, containing 0.05% Tween-20 (PBS-T) in the presence of 5% nonfat dry milk powder for 1 h at room temperature. The membranes were incubated overnight at 4°C with convalescent homologous PRRSV-specific pig antiserum obtained from a previous study [49] used at a 1/5000 dilution, or mouse monoclonal Abs specific to either adenovirus E1A (1/5000) (Millipore, Bedford, MA, USA), GFP (1/5000) (Roche) or GAPDH (1/10 000) (Sigma-Aldrich, St. Louis, MO, USA) proteins. Following incubation, membranes were washed three times with PBS-T and then incubated for 1 h at room temperature with anti-pig horseradish peroxidase (HRP)-conjugated IgG (Sigma-Aldrich) or anti-mouse-HRP-conjugated IgG (Bethyl, Montgomery, TX, USA). The signal was detected by enhanced chemiluminescence (ECL) (Perkin Elmer, Boston, MA, USA). The membranes were then exposed to Kodak Biomax Light-1 films.

### Recombinant GP5 production

Plasmid pGEX4T1 (Pharmacia Biotech, Piscataway, NJ, USA) encoding wild-type GP5 of the PRRSV IAF-Klop strain was obtained from a previous study [50]. Recombinant GP5 in fusion with Glutathione S-transferase (GST), designated hereafter rGP5, was produced in BL21 (DE3)pLysS competent *E*. *coli* cells (Promega, Madison, WI, USA) upon induction at OD_(600 nm)_ of 1.2 with 0.1 mM isopropyl β-D-1-thiogalactopyranoside (IPTG) for 4 h at 37°C. Bacterial cells were lysed by sonication in buffer (PBS, 0.5% Tween-20, 0.5% triton X-100, 0.5% NP40) and the whole bacteria protein extract was separated through 12% SDS-PAGE. The band corresponding to the rGP5 expected molecular weight was excised from the gel and electroeluted. The purified protein was dialyzed against PBS. The protein concentration was quantified with the DC protein assay kit as described above. The identity of the eluted protein was confirmed by Western blot using convalescent PRRSV-specific pig antiserum as above. The protein stock was then stored at −80°C for further use.

### Immunization of piglets and experimental challenge

Animal protocols were approved by the University’s and the Dairy and Swine Research and Development Centre’s (DSRDC) Animal Protection Institutional Committees according to the regulations of the Canadian Council for Animal Care. Seventy, three-week-old, Yorkshire-Landrace x Duroc pigs from the herd located at the DSRDC, Agriculture and Agri-Food Canada (Sherbrooke, QC, Canada) were used. At weaning, pigs at 21 days of age were divided into five groups of 14 pigs according to a randomized complete block design. The pigs were housed in the nursery rooms of the DSRDC swine complex. For each group, six pigs were kept separately for subsequent experimental challenge. Pigs were fed *ad libitum* with commercial non-medicated feed and had access to water. The swine herd was seronegative for PRRSV, transmissible gastroenteritis virus, *Mycoplasma hyopneumoniae* and *Actinobacillus pleuropneumoniae* prior to the experiment.

Pigs of three groups were immunized by both the intramuscular (IM) and intranasal (IN) routes at days 0 and 21 with 10^8^ TCID_50_ of rAdV expressing GFP (negative control), M-GP5 or M-GP5m. Pigs of group four were primed at day 0 IM and IN with 10^8^ TCID_50_ of rAdV expressing M-GP5m, and boosted at day 21 IM with 250 μg of rGP5 supplemented with 500 μg of QuilA (Brenntag Biosector, Frederikssund, Denmark), and mixed in equal volume with Freund’s incomplete adjuvant (Sigma-Aldrich). This group was designated M-GP5m/rGP5 hereafter. Pigs of group five were inoculated at days 0 and 21 IM with 2 mL of the commercial Ingelvac PRRSV MLV vaccine (Boehringer Ingelheim, St Joseph, MO, USA) inactivated 1 h at 56°C prior to the inoculation (designated hereafter as the inactivated vaccine). The inactivation process was conducted to avoid unwanted propagation of live PRRSV in the PRRSV-free swine herd of the research center. For IN immunization, pigs were sedated with stresnil (6–8 mg/kg of weight) given by the IM route. Blood samples were collected at 0, 21, 28, 35 and 49 days post-immunization (dpi). At 49 dpi, 8 pigs of each group were sedated and anesthetized (10 mg/kg of ketamine and 5 mg/kg xylazine) prior to being euthanized by exsanguination. The animal lungs were collected to perform bronchoalveolar lavage with 30 mL of PBS, pH 7.3.

The remaining pigs (*n* = 6) of each group were transferred at 42 dpi to the Faculty of Veterinary Medicine of the University of Montréal for experimental challenge. After an adaptation time of 7 days, the pigs were challenged (e.g. at 49 dpi) IN with 2 × 10^5^ TCID_50_ of the PRRSV FMV09-1155278 strain. The FMV09-1155278 strain was selected for experimental challenge on the basis of preliminary experiments in two pigs showing viremia from day 3 following infection with the virus. Animals were monitored daily for the presence of clinical signs of cough, dyspnea, diarrhea and inappetence. Blood samples were collected and rectal temperature was monitored at 0, 3, 5, 7, 10, 14 and 21 days post-challenge (dpc). Animals were weighed at days 0, 7, 14 and 21 dpc to determine the average daily body weight gain. At 21 dpc, pigs were euthanized to perform bronchoalveolar lavage with 30 mL of PBS as described above and for pathological examination. Determination of the percentage of macroscopic lung lesions, based on the consolidation of lung tissues, was determined according to a scoring system described elsewhere [51].

### Antibody response to PRRSV

#### Indirect ELISA

The presence of serum or bronchoalveolar lavage fluid (BALF) GP5-specific Abs was evaluated by an indirect ELISA using Immulon 2HB 96-well microtiter plates (Thermo Labsystems, Franklin, MA, USA). The plates were coated with 0.1 μg of rGP5 per well diluted in 0.05 M sodium carbonate buffered solution (pH 9.6) to a final volume of 100 μL. Following an overnight incubation at 4°C, the plates were washed 4 times with PBS-T and then saturated with 150 μL of PBS-T containing 1% bovine serum albumin (BSA) overnight at 4°C. One hundred μL of pig serum (used at a 1/200 dilution) or BALF diluted two-fold in PBS-T with 1% BSA were added into wells (in duplicate) and incubated for 2 h at 37°C. Plates were washed as described above and anti-pig HRP-conjugated IgG (used at a 1/10 000 dilution) or anti-pig HRP-conjugated IgA (used at a 1/25 000 dilution) (AbD Serotec, Raleigh, NC, USA) in PBS-T with 1% BSA were added for 1 h at 37°C. Plates were washed and the HRP signal was detected by adding 100 μL of tetramethylbenzidine (TMB, Sigma-Aldrich) per well. After an incubation of 20 min at room temperature, the reaction was stopped by adding 50 μL 1M H_2_SO_4_ to each well. Optical density (OD) was determined at 450 nm (using Tecan Infinite M1000 reader, Tecan Group Ltd, Männedorf, Switzerland). For each serum sample, the average OD was corrected by subtracting the OD of the uncoated well from the OD obtained with the antigen-coated well.

#### ELISA IDEXX

The IDEXX PRRS X3 HerdChek ELISA (IDEXX Laboratories, Westbrook, ME, USA) was used for detection of PRRSV-specific Abs in serum of animals. Serum samples with a calculated S/P ratio greater than 0.4 were considered positive as recommended by the manufacturer.

#### Serum neutralization assay

Serum or BALF samples were heat inactivated at 56°C for 30 min. Serial two-fold dilutions (starting at 1/2) of each sample were done in DMEM, and the neutralization test was performed by a viral cytopathic effect inhibition method using the IAF-Klop or FMV09-1155278 strain, 10^5^ cells/well of MARC-145 cells plated the day before the assay and four wells per specimen dilution [52]. The NAb titer was calculated at 96 h post cell infection and expressed as the reciprocal of the highest sample dilution neutralizing 100 TCID_50_ of the virus.

### Cellular immune response to PRRSV

Swine peripheral blood mononuclear cells (PBMC) from pigs to be challenged afterwards with PRRSV were isolated at 28, 35 and 49 dpi by density gradient centrifugation using Ficoll-Paque Plus (specific density of 1.077 g/mL; GE Healthcare, Piscataway, NJ, USA). PBMC were suspended in Roswell Park Memorial Institute 1640 medium (RPMI; Invitrogen) supplemented with penicillin (100 U/mL)/streptomycin (100 μg/mL) (Invitrogen), 10% FBS, 0.05 mM β-mercaptoethanol (Fisher Scientific, Nepean, Ontario, USA) and 10 mM 4-(2-hydroxyethyl)-1-piperazineethanesulfonic acid (HEPES, Invitrogen). PBMC were seeded at a concentration of 4 × 10^5^ cells per well in 96-well plates. Quadruplicate wells were exposed to 25 μL of either 200 TCID_50_ of heat-inactivated PRRSV IAF-Klop strain, cell culture medium (control cell cultures), or 1 μg/mL of Concanavalin A (Con A; Sigma-Aldrich) used as a positive control for lymphocyte functional activity. All cell cultures were incubated for 3 days at 37°C, and pulsed with 0.5 μCi of tritiated thymidine (specific activity, 6.7 Ci/mmol, Perkin Elmer) 18 h before harvesting cells onto a Wallac silica membrane (Perkin Elmer). The bound radioactivity was measured with Wallac Microbeta 1450 Trilux liquid scintillation counter (Perkin Elmer). The cell blastogenic responses were expressed by calculating the stimulation index (SI) which represents the ratio of the mean counts per minute (CPM) incorporated by the virus or mitogen-stimulated containing cell cultures to the mean CPM incorporated by the control cell cultures.

### RNA extraction and PRRSV real-time PCR

The QIAamp Viral RNA kit (Qiagen) was used to isolate viral RNA from the serum samples at 0, 5, 7 and 10 dpc as described in the manufacturer’s instructions. A commercial PRRSV real-time PCR diagnostic kit (NextGen, Tetracore Inc., Gaithersburg, MD, USA) was used for PRRSV quantification as recommended by the manufacturer. The quantification of PRRSV was determined by comparing the sample results with a standard curve based on the amount of serially diluted IAF-Klop strain produced in MARC-145 cells and titrated as TCID_50_/mL of viral particles in the MARC-145-infected cell culture supernatant [53]. The PRRSV qRT-PCR results were expressed in TCID_50_/mL of serum.

### Statistical analyses

Data were analyzed with a Proc MIX procedure of SAS (SAS Institute, Inc., Cary,NC, USA). Analysis of differences in Ab responses to GP5 in sera before and after challenge was performed with a Kruskal-Wallis test with Bonferroni adjustment. The other data were analyzed by ANOVA with Tukey adjustment.

## Results

### Expression of recombinant adenoviruses (rAdV)

**Figure 1:**
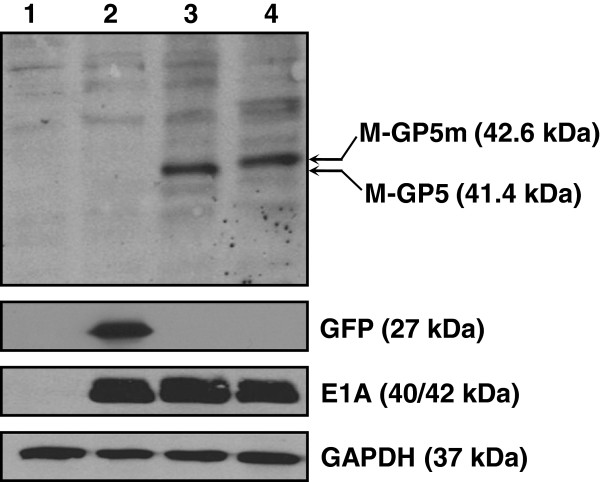
**Expression of recombinant adenoviruses (rAdV) in vitro.** Western blot analysis of A549 cell lysates from mock infected (lane 1) or infected with rAdV (MOI of 1) expressing GFP (lane 2), M-GP5 (lane 3) and M-GP5m (lane 4). Immunoblot was performed using GFP-specific mouse monoclonal antibody or a convalescent PRRSV-specific pig antiserum as primary antibody and an anti-mouse or anti-pig IgG-HRP as secondary antibody. Replication capability of rAdV was confirmed by E1A expression (lanes 2–4). Glyceraldehyde-3-phosphate dehydrogenase (GAPDH) immunostaining was used as a loading control.

Expression of proteins was detected by Western blot 24 h after infection of A549 cells with rAdV expressing GFP, M-GP5 or M-GP5m. As shown in Figure 1, GFP as well as M-GP5 and M-GP5m fusion proteins were expressed at the expected 27, 41.4 and 42.6 kDa molecular weights, respectively (lanes 2 to 4). E1A gene expression was confirmed in rAdV-infected cells, indicating the capability of rAdV to replicate in non trans-complementing cells.

### Antibody (Ab) response in immunized pigs

**Figure 2:**
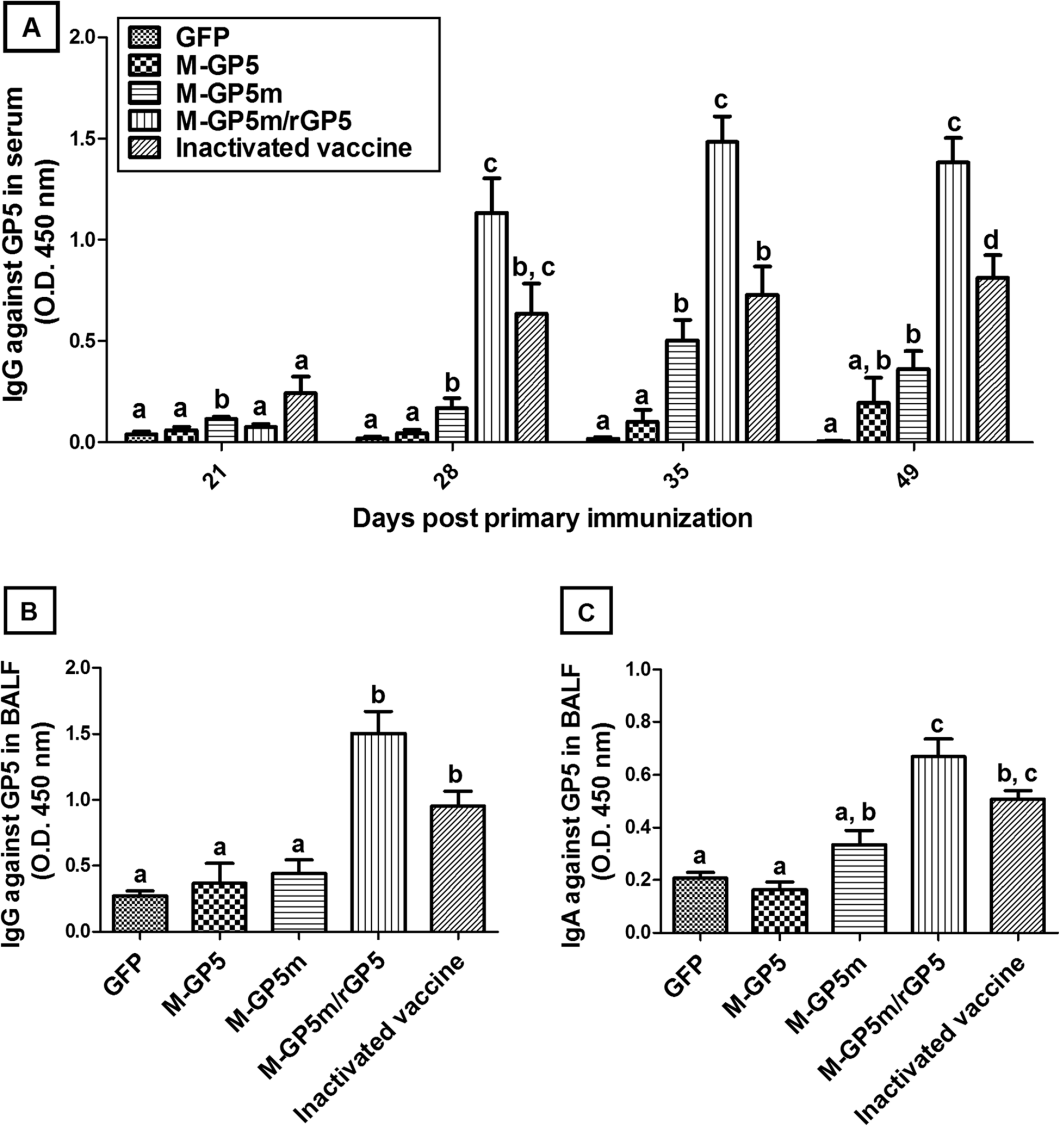
**Antibody responses in pigs vaccinated with the recombinant adenoviruses.** (**A**) IgG specific to GP5 were detected in serum samples of pigs (*n* = 14 per group) at various time points by indirect ELISA. Bronchoalveolar lavage fluid (BALF) of pigs were collected at 49 days post primary immunization and IgG (**B**) or IgAs (**C**) specific to GP5 were determined by indirect ELISA (*n* = 8 per group). Data are expressed as the mean + SEM. When two sets of data are labeled with superscripts of different letters, it indicates that these sets of data are statistically different (*P* < 0.01) for (**A**) or (*P* < 0.05) for (**B**) and (**C**).

As shown in Figure 2A, serum GP5-specific Abs were detected from 28 dpi in pigs immunized twice with M-GP5m-expressing rAdV (M-GP5m group), and to a much higher level in pigs immunized with either rAdV expressing M-GP5m followed by a boost with rGP5 (M-GP5m/rGP5 group) or twice with the inactivated vaccine. Significantly higher Ab response was observed in the M-GP5m/rGP5 group of pigs at 35 and 49 dpi when compared to the other groups of immunized pigs (*P* < 0.005). The M-GP5m group generated a better Ab response than that of the M-GP5 group at 28 (*P* < 0.01) and 35 dpi (*P* < 0.005). The M-GP5 group indeed showed the weakest Ab response among all groups of immunized pigs at any time point. There was no difference between the M-GP5m and inactivated vaccine groups at 28 and 35 dpi. In contrast, the Ab response observed in the inactivated vaccine group was higher (*P* < 0.005) than that of the M-GP5m group at 49 dpi.

The presence of GP5-specific IgG and IgA in BALF from 8 pigs of each experimental group was determined by indirect ELISA at 49 dpi (Figure 2B and C). The M-GP5m/rGP5 group developed higher GP5-specific IgG and IgA responses than the M-GP5 and M-GP5m groups (*P* < 0.005), being consistent with the serum results. However, the Ab response in the M-GP5m/rGP5 group was similar to that observed in pigs of the inactivated vaccine group. As observed with the serum results, pigs of the M-GP5 group showed the lowest GP5-specific Ab response in BALF.

The presence of NAb was not detected in sera or BALF of any of the immunized pigs, regardless of the immunization regime. Finally, it is noteworthy that pigs immunized with rGP5 did not develop Ab against GST at any time point of the experiment (data not shown).

### Cellular immune response in pigs following immunization

**Figure 3:**
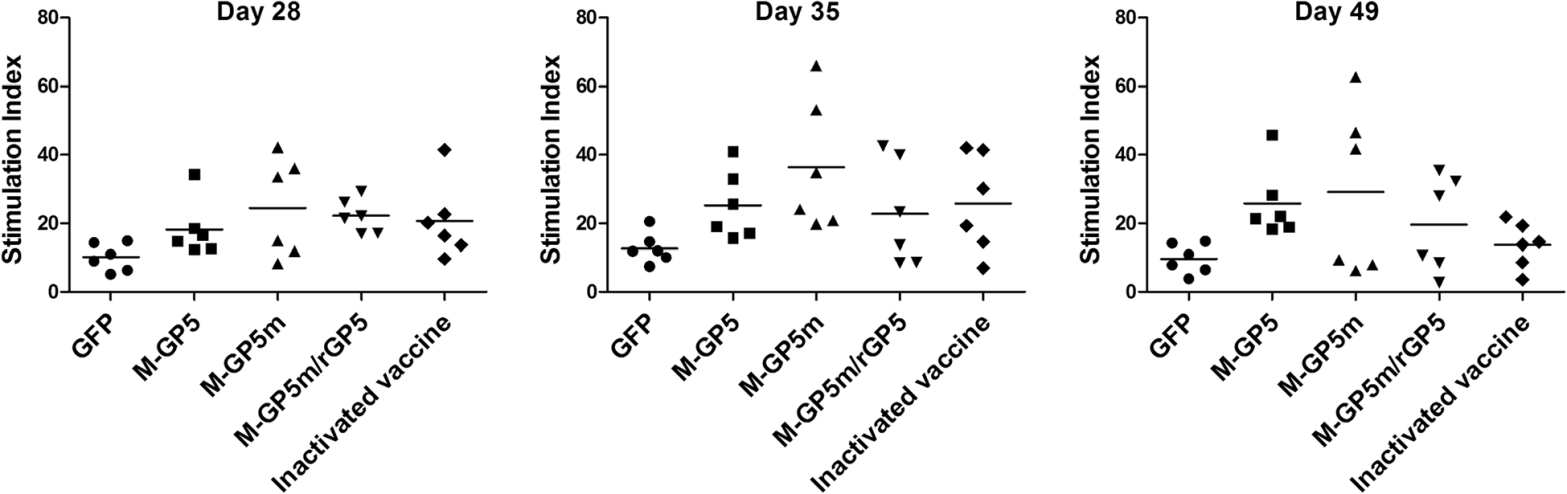
**Lymphocyte proliferative responses in pigs immunized with recombinant adenoviruses.** Peripheral blood mononuclear cells in quadruplicate were stimulated with inactivated PRRSV (MOI of 200) during 72 h. Cells were labeled with radioactive H^3^-thymidine incorporated DNA 18 h before cell harvesting. The data are expressed as stimulation index (SI) which represents the ratio of mean CPM of cells stimulated with the antigen divided by the mean of CPM of cells without antigen. Data represent the individual responses of each pig and the means of the group (*n* = 6 per group) at 28, 35 and 49 days post-immunization are represented by the horizontal bars.

The cell immune responses of six pigs per group that were used for experimental challenge were evaluated at 28, 35 and 49 dpi. As shown in Figure 3, no significant differences between the animal groups were observed due to the high variability in the cellular immune response obtained in individual pigs. Nonetheless, the SI mean in pigs of the M-GP5m group was higher than that obtained in the other groups at any time point following immunization.

### Protective efficiency against PRRSV challenge

#### Pathological signs and macroscopic lung lesions

One pig in the M-GP5m group suffered from posterior ataxia and was euthanized at 10 dpc. This animal was thus not considered in the challenge study. No clinical signs such as diarrhea, inappetence, cough or dyspnea were observed in any of the pigs throughout the experimental period of 21 dpc. The body temperature increased slightly at 3 dpc in all pigs but no other change was observed throughout the 21 dpc period. In addition, there was no difference in the average daily body weight gain among all groups of pigs. Percentages of macroscopic lung lesions were evaluated at 21 dpc. Pigs of the inactivated vaccine group showed the lowest macroscopic lung lesions (less that 1% in all pigs but one). There was no noticeable difference among all other groups of pigs regardless of the immunization regime with macroscopic lung lesions less than 5% (data not shown).

#### Antibody (Ab) response after experimental challenge

**Figure 4:**
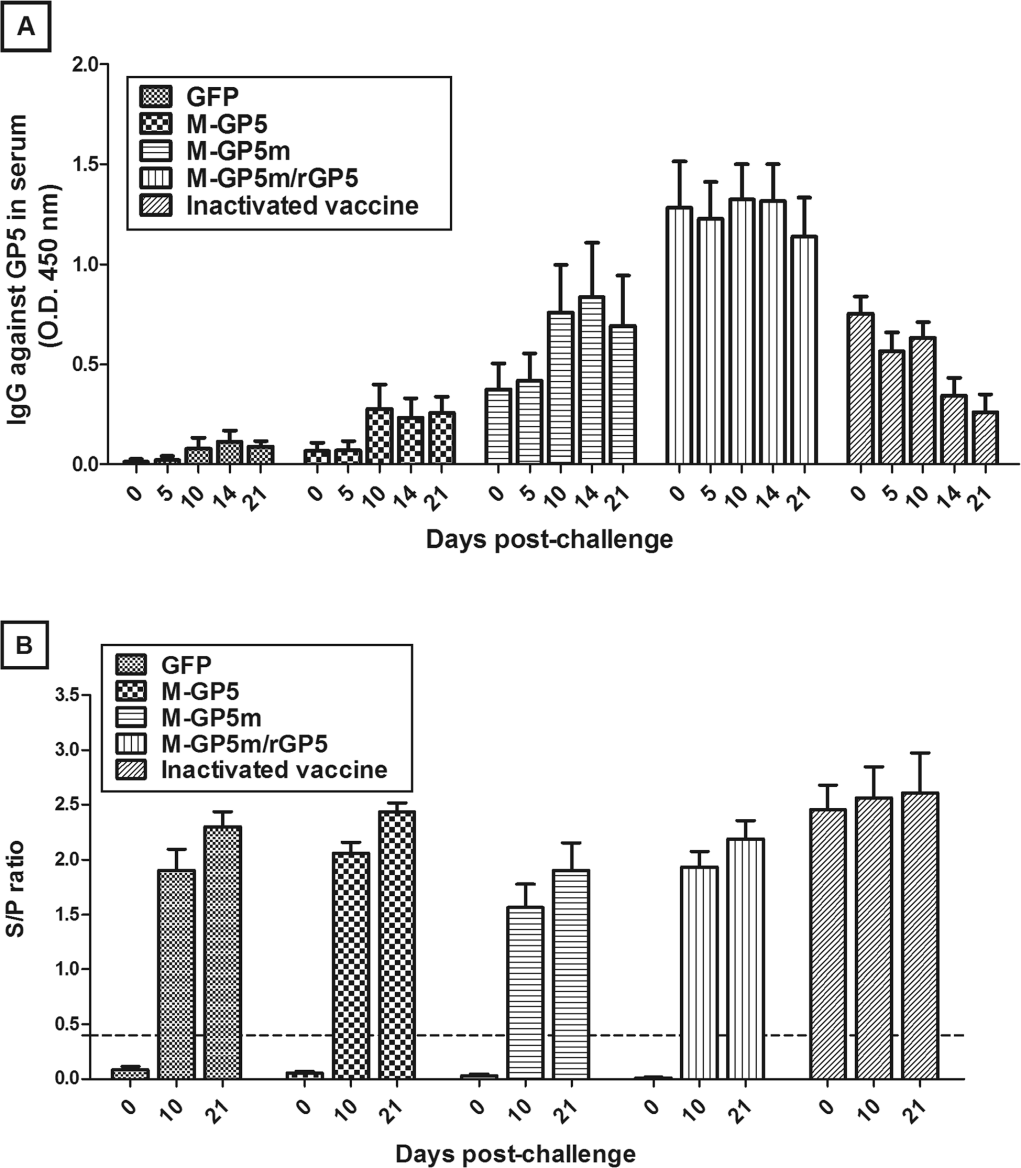
**Antibody responses in pig sera challenged with the PRRSV FMV09-1155278 strain.** (**A**) IgG specific to GP5 were detected in serum samples of pigs at various time points after challenge by indirect ELISA. Data are expressed as mean + SEM. (**B**) Kinetics of the IgG specific to PRRSV determined by HerdChek PRRS X3 ELISA are shown. Data are expressed in S/P ratio average. S/P ratio > 0.4 were considered positive (*n* = 6 pigs for all groups but the M-GP5m group with 5 pigs).

The results of serum GP5-specific Ab response after challenge are presented in Figure 4A. The GFP, M-GP5 and M-GP5m groups of pigs showed an increase, although not significant, in GP5-specific Abs at 10 dpc. The M-GP5m/rGP5 animal group had the same level of GP5-specific Abs before and after challenge whereas in the inactivated vaccine group there was a significant diminution of Ab level between 0 and 21 dpc (*P* < 0.05). As illustrated in Figure 4B, the level of serum Abs specific to PRRSV was also determined at 0, 10 and 21 dpc by the IDEXX ELISA. This ELISA detects Abs mainly specific to the PRRSV N protein [54,55]. Pigs that had not received the inactivated vaccine developed N protein-specific Abs from 10 dpc with no differences in these Ab levels between groups. As expected, pigs of the inactivated vaccine group showed a high level of anti-N Abs at 0 dpc.

As shown in Table 1, virus seroneutralization assays performed with the PRRSV IAF-Klop strain at 21 dpc indicate that all pigs of the inactivated vaccine group developed NAbs with a titer of 16 in 50% of the pigs. Two pigs in the M-GP5m/rGP5 group developed NAbs with titers of 2 and 4, respectively. No pigs from all other animal groups developed NAb response. When the assays were conducted with the FMV09-1155278 strain only pigs of the inactivated group developed NAbs at titers comparable to those obtained with the IAF-Klop strain (data not shown).

**Table 1:** PRRSV-specific neutralizing antibody titers in pigs at 21 day post challenge

**Group**	**< 2**^**a**^	**2**	**4**	**8**	**16**
**GFP**	6^b^	0	0	0	0
**M-GP5**	6	0	0	0	0
**M-GP5m**	5	0	0	0	0
**M-GP5m/rGP5**	4	1	1	0	0
**Inactivated vaccine**	0	0	2	1	3

^a^ Neutralizing antibody titer against the PRRSV IAF-Klop strain.

^b^ Number of pigs.

**Figure 5:**
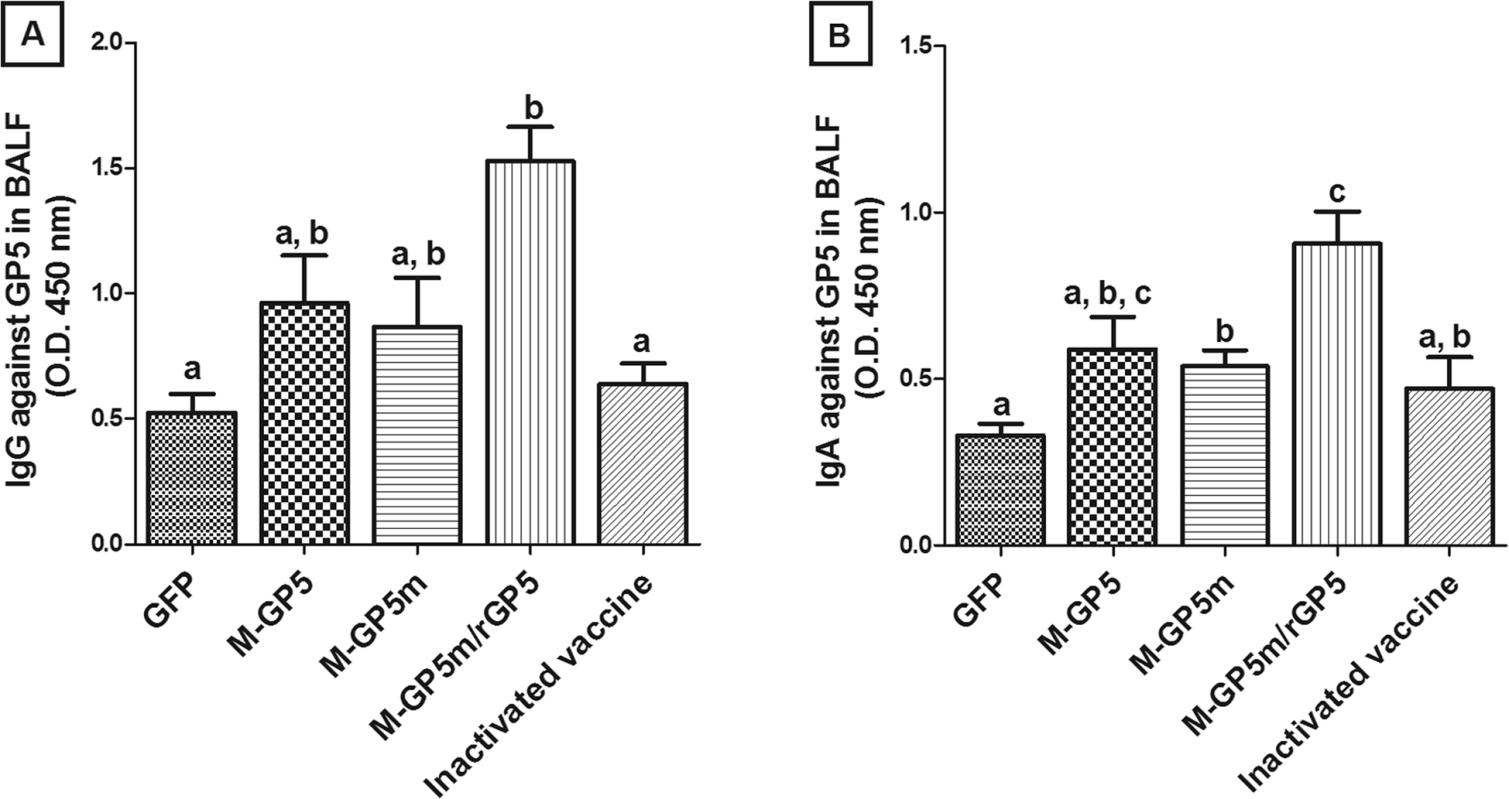
**Antibody responses in bronchoalveolar lavage fluids of pigs challenged with the PRRSV FMV09-1155278 strain.** Indirect ELISA was performed to detect IgG (**A**) and IgA (**B**) specific to GP5 of pigs at day 21 post-challenge. Data are expressed as the mean + SEM (*n* = 6 pigs for all groups but the M-GP5m group with 5 pigs). When 2 sets of data are labeled with superscripts of different letters, it indicates that these sets of data are statistically different (*P* < 0.05).

The presence of GP5-specific IgG and IgA in BALF was determined at 21 dpc (Figure 5A and B, respectively). Pigs of the M-GP5m/rGP5 group developed higher (*P* < 0.0001) GP5-specific IgG response than animals of the GFP and inactivated vaccine groups. This response was slightly better but not significantly different from that observed in the M-GP5 and M-GP5m groups of pigs. There was also no significant difference in the GP5-specific IgG level between the GFP, M-GP5, M-GP5m and the inactivated vaccine pig groups. However GP5-specific IgA level produced in pigs of the M-GP5m/rGP5 group was significantly higher than that observed in all other groups of animals (*P* < 0.05) except the M-GP5 group. None of the pigs developed NAbs in BALF following PRRSV challenge.

### Viremia

**Figure 6:**
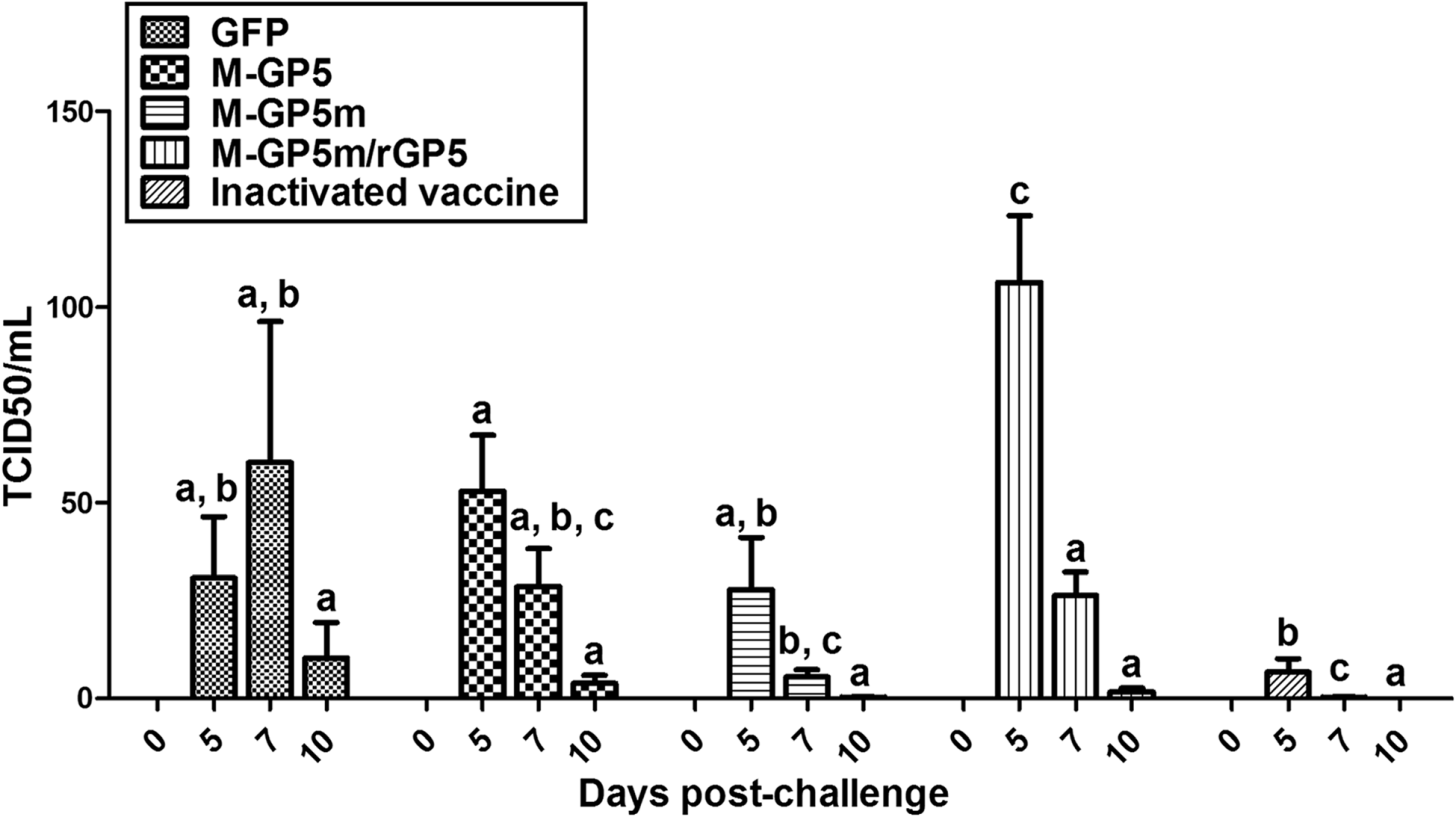
**Viremia of pigs immunized with recombinant adenoviruses and challenged with the PRRSV FMV09-1155278 strain.** Pigs were challenged intranasally with 2 × 10^5^ TCID_50_ of PRRSV FMV09-1155278 strain at day 49 post primary immunization. The PRRSV loads were determined at 0, 5, 7 and 10 days post-challenge by quantitative real-time RT-PCR and expressed as TCID_50_/mL. Data are expressed as the mean + SEM (*n* = 6 pigs for all groups but the M-GP5m group with 5 pigs). When 2 sets of data are labeled with superscripts of different letters at the same sampling day, it indicates that these sets of data are statistically different (*P* < 0.05).

Viremia was monitored in sera of pigs at 0, 5, 7 and 10 dpc (Figure 6). The viremia level of the GFP group increased between 5 and 7 dpc, and was still detectable at 10 dpc (with a mean of 10.36 in TCID_50_/mL). The mean level of viremia of the M-GP5 group was not different when compared to the GFP group at 5 dpc, but decreased from 5 dpc to a mean level of 3.86 TCID_50_/mL at 10 dpc. The viremia level in pigs of the M-GP5m group at 5 dpc was somewhat lower than that of the GFP and M-GP5 groups and thereafter decreased to nearly undetectable levels at 10 dpc (mean of 0.20 TCID_50_/mL). Pigs of the inactivated vaccine group showed the lowest viremia levels among pigs of all other groups. The viremia level was almost undetectable at 7 dpc (mean of 0.21 TCID_50_/mL) and undetectable at 10 dpc. In contrast, pigs of the M-GP5m/rGP5 group showed the highest viremia level (mean of 106.42 TCID_50_/mL) at 5 dpc when compared to that of all other animal groups at 5 dpc (*P* < 0.05). The viremia level in the M-GP5m/rGP5 group decreased at 7 dpc but still was higher than those of pigs of the M-GP5m and inactivated vaccine groups (*P* < 0.05). Viremia in this group was still detectable with a mean of 1.72 TCID_50_/mL at 10 dpc.

## Discussion

In this study, replicating but nondisseminating human adenoviruses serotype 5 were used to express PRRSV proteins within the pig allowing induction of a PRRSV-specific immune response. Pigs immunized with rAdV expressing M-GP5m show higher systemic and mucosal GP5-specific Ab responses than those of the M-GP5 group using an ELISA. Theoretically, mutations in GP5m were expected to generate a better NAb response than the use of WT GP5. The first mutation introduced in GP5m abolishes the decoy epitope to which a robust non NAb response has been associated [8]. Moreover, abolishing the N30 glycosylation site through the same mutation was also expected to generate a better NAb response [38]. Insertion of the PADRE sequence between the decoy and neutralizing epitopes was also expected to generate better NAb and T-cell responses as reported elsewhere [23,25]. Finally abrogation of the N51 glycosylation site was also expected to enhance NAb production [11,56]. Similarly to pigs of the M-GP5 group, NAbs were not detected in pigs of the M-GP5m group, nor during the immunization phase nor following PRRSV experimental challenge. This result was in contrast with that reported by Li et al. where immunization of pigs with a plasmid vector expressing a GP5 protein with similar mutations e.g. insertion of the PADRE sequence and abrogation of the N30 and N51 glycosylation sites resulted in significant and higher NAb response than that observed in animals immunized with a native form of GP5 [22]. Although no NAbs were detected, a decrease in serum viral load with no complete elimination of the virus was observed in challenged pigs of both the M-GP5 and M-GP5m groups at 7 and 10 dpc when compared to the control GFP group. This decrease in virus load might be due to higher mean SI in both of these groups when compared to the mean SI of all other pig groups before challenge (Figure 3). The virus load decrease was even more pronounced in pigs of the M-GP5m group which showed the highest mean SI at any time point before challenge. These results might indicate a role of the cellular immune response in virus clearance as suggested elsewhere [57-59]. However, caution must be made with this interpretation as pigs of the inactivated vaccine group showed the lowest viremia levels and SI values in this study.

Pigs of the M-GP5m/rGP5 group showed the highest Ab response among all experimental animal groups. This strong response was observed from one week (e.g. at 28 dpi) following inoculation with rGP5. This was likely due to the prime-boost regimen used since single inoculation of animals with rGP5 generated an Ab response only from day 14 after immunization and yet at a much lower level than that in pigs of the M-GP5m/rGP5group (data not shown). However, pigs of the M-GP5m/rGP5group showed, following challenge, higher viremia levels than the control group, indicating that the high Ab level generated in these pigs did not provide protection whatsoever but instead appeared to increase viremia. This increase in viral load can be explained by the antibody-dependent enhancement (ADE) mechanism by which non NAb mediate the attachment of PRRSV to monocyte/macrophage target cells via the Ab Fc receptor at the surface of these cells, resulting in cell internalization of the virus, and thereof, increased virus replication [60]. Indeed, the ADE mechanism was suggested to be associated with PRRSV GP5 decoy epitope and N protein [61-63]. Further studies are needed to determine whether the use of rGP5 devoid of a functional decoy epitope would impact the presumably-ADE associated viremia level in immunized pigs following experimental challenge with PRRSV.

Animals of the inactivated vaccine group developed systemic and mucosal Ab responses. These immune responses were higher, albeit to non significant levels, than the M-GP5m during the immunization phase. IgA and IgG were detected in the lung lavage of animals of the inactivated vaccine group that were immunized through the systemic IM route in contrast to animals of the M-GP5m group that were vaccinated by both the mucosal (IN) and systemic (IM) routes. The presence of Ab in the lung lavage of pigs of the inactivated vaccine group is likely due to the passage of serum Ab through the lung mucous membranes by passive transudation [64]. Whatever the mechanism is, all pigs of this group developed significant amounts of serum NAb after challenge and showed the lowest percentages of gross lesions and viral load when compared to pigs of all other groups. Although the role of NAb for in vivo protection against PRRSV is still a debate [65], a minimal serum NAb titer of 8 comparable to what was obtained in our pigs was reported to be necessary to prevent viremia [66,67].

In this study, pigs were challenged with a virus strain heterologous to that used for the immunization phase due to our difficulty in reproducing clinical disease and viremia with the IAF-Klop strain. Thus the FMV09-1155278 PRRSV strain that was recently isolated in Québec was used for experimental challenge. Moreover the utilization of a heterologous virus strain for challenge was of interest as it is well known that PRRSV antigenic variation occurs under field conditions [68]. It is noteworthy that the PRRSV FMV09-1155278 strain has the same GP5 neutralizing epitope sequence (SHLQLIYNL) as the VR2332 strain-based PRRSV MLV commercial vaccine used in this study in an inactivated form. This sequence differs by two aa (in bold and underlined) when compared to the IAF-Klop neutralizing epitope (S**Q**LQ**S**IYNL). Thus, NAb titers were expected to be higher in pigs of the inactivated vaccine group using a homologous viral strain in the virus seroneutralization test. However, the fact that comparable NAb titer results were obtained in pigs of the latter group using either PRRSV IAF-Klop or FMV09-1155278 strain in the assay might suggest that these aa are not critical for the GP5 neutralizing epitope. Alternately, the results may reflect different susceptibility of the virus strains to in vitro neutralization [69].

No clinical signs were observed in the virus-exposed pigs including control animals indicating that the PRRSV FMV09-1155278 strain used for challenge was attenuated. This might be attributed to the serial passage of the virus on MARC145 cells. Despite the absence of clinical signs, a viremia state, a parameter used in other studies to ascertain protection in immunized pigs with or without overt clinical signs following challenge [15,24,70], was observed in all control animals at 5 dpc. In addition, pigs of all groups developed an Ab response specific to the PRRSV N protein at 10 dpc indicating productive infection in these animals.

In summary replicating but nondisseminating adenoviruses expressing M-GP5 or M-GP5m conferred partial protection against PRRSV. The degree of immune protection was better in the case of M-GP5m expression. Our data were consistent with those of other vector-based vaccination strategies proposed to date that have generated only partial protection of animals.

## Competing interests

The authors declare that they have no competing interests.

## Authors’ contributions

ER performed the experiments, analyzed the data and wrote the paper. AG was involved in the lymphoproliferation assays and in the in vivo work. M-CS-L produced the recombinant adenoviruses. BM developed the recombinant adenoviruses system and was involved in editing the manuscript. CAG supervised, coordinated and conducted the in vivo work for the experimental challenge. ML supervised, coordinated and conducted the in vivo work in the immunization experiments. DA conceived and designed the vaccination strategy, supervised the project and edited the manuscript. All authors discussed the results and critically revised and approved the final manuscript.
